# Primary retroperitoneal mucinous cystadenoma in a female patient: A case report

**DOI:** 10.1016/j.ijscr.2022.107099

**Published:** 2022-04-20

**Authors:** Ali Taherinezhad Ledari, Ghodsieh Kamrani, Tina Rouhi, Novin Nikbakhsh

**Affiliations:** a Student research committee, Babol University of Medical Sciences, Babol, Iran; b Clinical Research Development Center, Shahid Beheshti Hospital, Babol University of Medical Sciences, Babol, Iran; c Cancer Research Center, Pathology Department, Babol University of Medical Sciences, Mazandaran, Iran; d Associate Professor of Thoracic surgery, Cancer Research Center, Babol University of Medical Sciences, Mazandaran, Iran

**Keywords:** Retroperitoneal cyst, Cystadenoma, Case reports

## Abstract

**Introduction:**

Primary retroperitoneal mucinous cystadenoma is an extremely uncommon tumor occurring mostly in females. The histogenesis of PRMC remains unclear and Open surgery is the most impressive treatment.

**Case presentation:**

We present a 20-year-old Iranian woman with history of intermittent abdominal pain. In physical examination, her abdomen had a mildly asymmetrical distention and a round shape mass was palpated in right abdomen also she had a mild tenderness in right abdomen. Radiologic assessment revealed a right retroperitoneal smooth cystic mass (20 × 15 cm) without invasive features. The patient underwent complete surgical excision of the tumor by a laparotomic approach because of its size. The lesion was gently dissected from the contiguous organs and removed completely without spillage of its content. In microscopic investigations, sections showed a unilocular cyst with fibrous wall lined via monolayered bland looking columnar mucinous epithelium and no atypia or stromal invasion was presented. Diagnosis of a PRMC was made. The patient has discharged without any complications in 2 days' postoperative course.

**Discussion:**

Generally, cysts are asymptomatic and are found fortuitously after a routine checkup assessment. Symptoms and signs related to this cyst are mainly because of their huge size and compression effect. Radiological assessments are effective in specifying the lesion preoperatively; although, the final diagnosis always needs histopathological verification.

**Conclusion:**

When the tumor is diagnosed, full resection should be considered because of the infectious or malignant potential of the tumor.

## Highlights

• Full resection of retroperitoneal mucinous cystadenoma because of the infectious or malignant potential of the tumor
• Histological and macroscopical likeness of primary retroperitoneal cystadenoma to ovarian mucinous cystadenoma
• CK7 and CA125 positivity in retroperitoneal mucinous cystadenoma because of its Mullerian origin
• Symptoms and signs related to the cyst mainly because of its huge size and compression effect

## 1 Introduction

Primary retroperitoneal mucinous cystadenoma (PRMC) is a rare tumor occurring in the retroperitoneum mainly in females. Less than 100 cases of PRMC have been reported since 1965. The histogenesis of PRMC remains unclear because no epithelial tissue initially exists in the retroperitoneum. Primary symptom is usually non-specific abdominal pain due to its mass result, although in early stages, PRMC is often asymptomatic [1]. The diagnosis is specified via pathological findings instead of radiological findings; thus, surgery is necessary for both diagnosis and treatment. Surgery resection of PRMC has increasingly been used even if radiological signs, such as calcification and enhanced mural nodule, demonstrate malignancy. Open surgery remains the most impressive treatment however successful laparoscopic resection has also been reported [2], [3]. In this study, we reported a case of the successful open surgery of PRMC and review the literature. This work has been reported in line with the SCARE criteria [9].

## 2 Presentation of the case

Our patient was a 20-year-old woman without any past medical history. She complained of right-sided intermittent abdominal pain that started 2 months before her first Reference to our hospital. She enters to the study after signing the informed confirmation. There wasn't any history of benign or malignant abdominal tumors in her family history. In physical examination, vital signs including BP: 110/70 HR: 85 RR: 18 T: 36.7, her abdomen had a mildly asymmetrical distention and a round shape mass was palpated in right abdomen also she had a mild tenderness in right abdomen. Pelvic examination was normal. She was in fine general status.

**Fig. 1:**
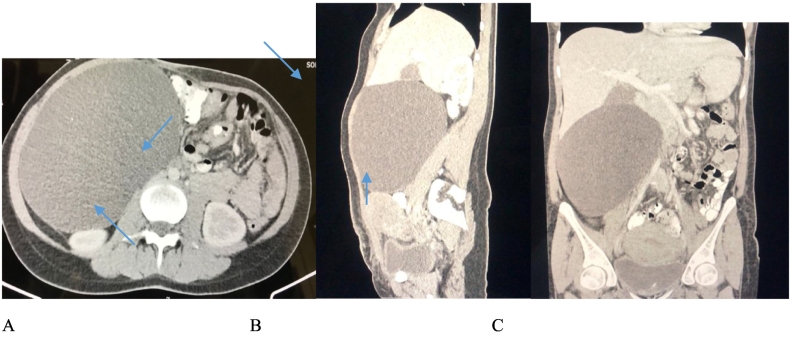
(A) Coronal view abdominopelvic computed tomography scan (CT scan) indicated a homogeneous unilocular cystic mass in the retroperitoneal space measuring about 20 × 15 cm with no enhancement and solid part in right quadrant of abdomen (B) sagittal view (C) axial view.

**Fig. 2:**
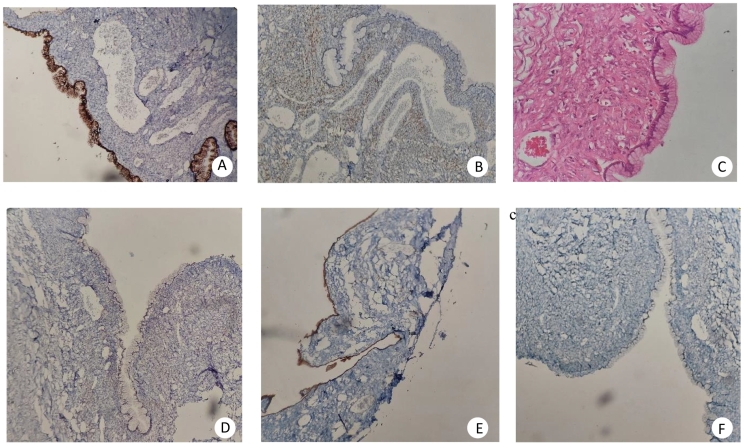
(A) Positive for CK7 (B) Immunochemistry for ER was negative in the epithelial lining and positive in stromal cells (C) Unilocular cyst with fibrous wall lined via monolayered bland looking columnar mucinous epithelium and no atypia or stromal invasion was presented (D) Negative for CK20 (E) Positive for CA125 (F) Negative for CDX2.

In laboratory examinations, complete blood count and serum chemistry were all within normal ranges. Tumor markers containing carbohydrate antigen 19-9 (CA19-9), cancer antigen 125 (CA 125), and carcinoembryonic antigen (CEA) were within normal ranges. An ultrasound study indicated a large cystic lesion about 16 × 15 cm with regular limits in the subhepatic and anterior region of the right kidney. Abdominopelvic computed tomography scan (CT scan) indicated a homogeneous unilocular cystic mass in the retroperitoneal space measuring about 20 × 15 cm with no enhancement and solid part in right quadrant of abdomen (Fig. 1A). The right kidney and ascending colon were displaced medially via the lesion (Fig. 1B). The feature was matchable with a large retroperitoneal cyst. The patient underwent complete surgical excision of the tumor by a laparotomic approach because of its size. A massive retroperitoneal cystic mass was found with mild adhesions to the ascending colon and anterior region of the right kidney. The lesion was mildly dissected from the contiguous organs and removed completely without spillage of its content. Appendix, uterus, ovaries, fallopian tubes, and pancreas seemed normal in appearance, location, and size. Also, the cyst contained clear serous fluid. In microscopic investigations sections showed a unilocular cyst with fibrous wall lined via monolayered bland looking columnar mucinous epithelium and no atypia or stromal invasion was presented (Fig. 2). Also, sections showed neutrophilic infiltration in serosal layer with congested vessels. Immunochemistry for CK7 and CA125 was positive which shows its Mullerian origin, whereas, for CDX2 and CK20 was negative that rule out its origin from gastrointestinal system [10]. Also, immunochemistry for ER was negative in the epithelial lining and positive in stromal cells (Fig. 2). Diagnosis of a PRMC with mullerian origin was made. The patient has discharged without any complications in 2 days postoperative course.

## 3 Discussion

Primary retroperitoneal mucinous cystadenomas (PRMCs) are extremely uncommon tumor and they show histological and macroscopical likeliness to ovarian cystadenomas. They could be found in each section of the retroperitoneum with no adhesion to the ovary [4]. Two main hypotheses have been proposed for the histogenesis of PRMC. First, they are thought to arise from heterotopic ovarian tissue because of its likeliness to ovarian mucinous cystadenomas. Estrogen receptor positivity in stromal cells of PRMCs has also been indicated. Second, these tumors arise from an invagination of peritoneal mesothelium that is entrapped and undergoes mucinous metaplasia and cyst formation [5], [6]. Generally, cysts are asymptomatic and are found fortuitously after a routine checkup assessment. Symptoms and signs related to this cyst are mainly because of their huge size and compression effect on contiguous organs. This pathology generally happens in females and most of them complaint about ambiguous abdominal discomfort [7]. Its etiology is not clear. Finding a retroperitoneal cyst in sonography mandates advance imaging modality. CT scan is the gold standard radiologic assessment for diagnosis and finding many aspects of retroperitoneal cysts including size of the cyst, wall thickness, location, presence of calcifications, enhancement pattern, cyst wall regularity, invasion of the contiguous organs, myxoid stroma, fat, and necrosis [8]. Radiological assessments are effective in specifying the lesion preoperatively; although, the final diagnosis always needs histopathological verification. Tumor markers such as AFP, CA 125, CEA, and CA 19-9 are in the usual range in these patients and wouldn't aid us in diagnosis and follow up. when the tumor is diagnosed, full resection should be considered because of the infectious or malignant potential of the tumor. An exploratory laparotomy via full enucleation is the usual treatment. In the present case, we decided to perform an open laparotomy exploration. It is main to note that cystic fluid spillage has to be intercepted because of the unclear pathology of the lesion in most of the cases [6]. According to a review of the cases reported in the literature, these tumors are classified into mucinous cystadenomas, mucinous borderline tumors of low malignant potential, and mucinous cystadenocarcinoma. The most prevalent type is the retroperitoneal mucinous cystadenoma. A huge, multilocular or unilocular benign cystic tumor which is not related with recurrence after surgery. This was the type that our patient introduced. In another type, the cells of the inner lining present with foci of epithelial proliferation. This type is similar to the ovarian mucinous cystadenomas of low malignant potential. The third type is the mucinous cystadenocarcinoma that is an invasive tumor [5], [6].

## 4 Conclusion

In brief, this case reports a successful surgical management of PRMC. We advocate that patients presenting with this uncommon tumor should undergo prompt investigation and their management is foremost decided via expert group after exact review of their preoperative radiological findings. When the tumor is diagnosed, full resection should be considered because of the infectious or malignant potential of the tumor. An exploratory laparotomy with full enucleation is the usual treatment. It is important to note that cystic fluid spillage has to be intercepted because of the unclear pathology of the lesion in most of the cases.

## Sources of funding

This research did not receive any specific grant from funding agencies in the public, commercial, or not-for-profit sectors.

## Ethical approval

This study was approved by ethical committee of Babol University of Medical Sciences.

## Consent

Written informed consent was obtained from the patient for publication of this case report and accompanying images. A copy of the written consent is available for review by the Editor-in-Chief of this journal on request.

## Author contribution

Ghodsieh Kamrani: Detected the case and lead the study.

Ali Taherinezhad Ledari: Wrote the manuscript, collected data.

Tina Rouhi: Revised the manuscript.

Novin Nikbakhsh: Did the surgery.

## Research registration

None.

## Guarantor

Ali Taherinezhad Ledari.

## Provenance and peer review

Not commissioned, externally peer-reviewed.

## Declaration of competing interest

There was no conflict of interests in this study.

## References

[1] Lee S.Y., Han W.C. (2016). Primary retroperitoneal mucinous cystadenoma. Ann. Coloproctology.

[2] Lee S.E., Oh H.C., Park Y.-G., Choi Y.S., Kim M.K. (2015). Laparoscopic excision of primary retroperitoneal mucinous cystadenoma and malignant predicting factors derived from literature review. Int. J. Surg. Case Rep..

[3] Afzal Z., Stupalkowska W., Mahler-Araujo M.B., Bowden D., Davies R.J. (2020). A case of successful surgical management of primary retroperitoneal mucinous cystadenoma. J. Surg. Case Rep..

[4] Jai S.R., Bouffetal R., Chehab F., Khaiz D., Bouzidi A. (2009). Primary retroperitoneal mucinous cystadenoma. Arch. Gynecol. Obstet..

[5] Lung J., Gracey A., Rosales A., Bashover E., Sbar A., Nazim M.H. (2019). Laparoscopic excision of a retroperitoneal mucinous cystic neoplasm: a case report. Int. J. Surg. Case Rep..

[6] Nardi W.S., Dezanzo P., Quildrian S.D. (2017). Primary retroperitoneal mucinous cystadenoma. Int. J. Surg. Case Rep..

[7] Fujita N., Nishie A., Asayama Y., Kiyoshima K., Kubo Y., Honda H. (2012). A male case of primary retroperitoneal mucinous cystadenoma: a diagnostic dilemma. Jpn. J. Radiol..

[8] Foula M.S., AlQattan A.S., AlQurashi A.M., AlShaqaq H.M., Gari M.K.M. (2019). Incidentally discovered huge retroperitoneal mucinous cystadenoma with successful laparoscopic management: case report. Int. J. Surg. Case Rep..

[9] Agha R.A., Franchi T., Sohrabi C., Mathew G., for the SCARE Group (2020). The SCARE 2020 guideline: updating consensus Surgical CAse REport (SCARE) guidelines. Int. J. Surg..

[10] Selves J., Long-Mira E., Mathieu M.C., Rochaix P., Ilié M. (2018 Apr 5). Immunohistochemistry for diagnosis of metastatic carcinomas of unknown primary site. Cancers (Basel).

